# Identification of Fungi Isolated from Oral Cavity of Patients with HIV Using MALDI–TOF MS

**DOI:** 10.3390/jcm10081570

**Published:** 2021-04-08

**Authors:** Zuzanna Pawlak, Szymon Andrusiów, Magdalena Pajączkowska, Adriana Janczura

**Affiliations:** 1 Students Scientific Society of Infectious Diseases, Liver Diseases and Acquired Immune Deficiencies, Faculty of Medicine, Wroclaw Medical University, 51-149 Wroclaw, Poland; z.pawlak95@gmail.com (Z.P.); szymon0946@wp.pl (S.A.); 2 Department of Microbiology, Faculty of Medicine, Wroclaw Medical University, 50-368 Wroclaw, Poland; magdalena.pajaczkowska@umed.wroc.pl

**Keywords:** *Candida*, MALDI–TOF MS, HIV, oral cavity fungal flora

## Abstract

Background: A growing incidence of invasive fungal infections, especially among immunocompromised patients, has given increased significance to microbiological diagnostics of yeast-like fungi. More accurate and faster fungi identification methods that can compete with classical methods are being searched for. In this paper, classical microbiological methods are compared to MALDI–TOF MS (matrix-assisted laser desorption/ionization time-of-flight mass spectrometry). Methods: The diagnostic material was collected from buccal mucosa from 98 adults, including 69 with HIV. Only positive cultures were included in the study. Results: Matching results were obtained in 45 samples, and there were nonmatching results in 35 samples, with the majority of these in the study group, constituting 50% of identifications within this group. A particularly common mistake resulting from the use of classical methods is the false identification of *C. dubliniensis* as *C. albicans.* Additionally, *C. tropicalis* proves to be difficult to identify. Conclusions: Our results and literature data suggest that MALDI–TOF MS should be considered an effective alternative to classical methods in terms of fungi identification, especially among HIV-positive patients, due to the different morphology of fungal colonies.

## 1. Introduction

Fast and accurate identification of pathogenic microbes is crucial for effective treatment. *Candida albicans* remains the most important species among yeast-like fungi pathogenic to people. This species colonizes mucosal surfaces of the human body and is also isolated from infections. Identification of *C. albicans* from clinical material is usually unambiguous, with plenty of methods allowing for a fast and accurate identification. Nowadays, it has become more common for other *Candida* species (*Candida* non*-albicans*) to be isolated from patients. These microorganisms can be more invasive and less susceptible to antifungal drugs compared to *C. albicans* [1]. Furthermore, the difficulties associated with the identification of these species can result in mistakes and the possibility of obtaining the wrong results of microbiological tests, subsequently leading to incorrect treatments of patients. Additionally, classical microbiological methods used to identify yeast-like fungi are time-consuming and based on matching cultures’ and strains’ individual characteristics to specific species. Therefore, there is a need for alternative methods to hasten the diagnostics and make the results more reliable than those produced by classical methods used in most laboratories across the world [2].

Immunocompromised patients, including HIV-infected patients, are a special group prone to fungal infections. Fast and precise diagnosis is extremely important in this group as it allows doctors to introduce the correct treatment and minimizes the risk of generalization of the infection [3]. Difficulties in the identification of microorganisms from HIV patients are observed when classical microbiological methods are used. It is possible that there are some morphological differences between fungal colonies isolated from HIV patients and healthy people [4]. As they rely on a morphological assessment of colonies on several types of media, classical microbiological methods strongly depend on the microbiologists’ experience. That is why it could be more effective to leverage more objective methods such as PCR (polymerase chain reaction) or MALDI–TOF MS (matrix-assisted laser desorption/ionization time-of-flight mass spectrometry) in clinical practice more often. Additionally, MALDI–TOF MS requires less time to perform (10–15 min) compared to classical methods, which can take a few days [5,6].

Usually, the first diagnostic step of classical methods is the preparation of a direct smear using material from patients (all except feces). Microscopic assessment of clinical material is often sufficient to confirm a fungal presence, make a preliminary determination of the genus and the approximate quantity. In the case of the oral cavity, a smear can be taken and dyed using the Gram method, with a fungal presence yielding a dark purple color in the aforementioned test [7].

Most fungi pathogenic to humans are grown on the Sabouraud solid medium. This medium is enriched with substances such as chloramphenicol or gentamicin to inhibit bacterial growth and is used to isolate fungi. Sometimes, it is also useful to choose a liquid medium—Sabouraud broth.

Culturing time depends on the fungal species. Incubation is sufficient after 48–72 h, but it is typically prolonged to 7 days. The optimal temperature for the growth of a pathogenic fungal culture is 30 °C.

Several types of media are used to identify grown cultures, allowing us to

- assess the colony macroscopically—Sabouraud medium; potato or corn medium.
- assess the fungi (mycelium, hyphae, chlamydospores) microscopically—rice medium; corn medium.
- assess the biochemical features of the fungi—commercial chromogenic media (ChromAgar *Candida* Becton Dickinson, Chromogenic *Candida* Selective Agar ARGENTA).

Sabouraud media enable a macroscopic assessment of colony morphology, including shape, surface, color, and edges. Furthermore, the rice medium leveraging the Dalmau technique enables culture fungi to create mycelium and pseudomycelium. Fungi grow in clusters of oblong cells, along which aciniform or oviform yeast cells can be found, or create chlamydospores placed on the main hyphae and side branches [7].

The chromogenic medium is designed for the initial identification of *C. albicans*, *C. glabrata*, *C. krusei*, and *C. tropicalis* species [8]. This medium type makes it possible to distinguish fungi based on their morphology (e.g., edges of the colony, different from the center) and color (e.g., from green through purple to white). The colonies’ color depends on the enzymatic activity of the fungi. During the degradation of chromogenic substrates, the enzymes released will color the compounds. *C. albicans* and *C. dubliniensis,* two closely related fungi, produce hexosaminidase, while *C. glabrata, C. tropicalis,* and *C. krusei* produce fosfatase [8,9]. Furthermore, colonies cultured on isolating media can be assessed under the microscope using lactophenol blue (Cotton-Blue), with fungi obtaining the same color.

Another method that allows fungal identification is culturing in different temperatures. This method is particularly useful for the differentiation between *C. albicans* and *C. dubliniensis*. These species are difficult to distinguish using the macroscopic method (both look smooth and creamy), the microscopic method (both produce chlamydospores), or the biochemical method (on CHROMagar, *Candida*–*C. dubliniensis* is dark green while *C. albicans* is green). Unlike *C. albicans*, *C. dubliniensis* grows poorly at 42 °C and does not grow at all at 45 °C. In some cases, the previously described classical microbiological methods are not sufficient to ensure the accuracy of identification, implying the need to use alternative methods.

MALDI–TOF MS is a method that uses mass spectrometry to define the NMR (nuclear magnetic resonance) spectrum of genus-specific proteins and compares this with a database. Ribosome proteins play a significant role in this method because they are usually specific to the genus [10]. The sample with the microorganism is mixed with a matrix solution, and then laser ionization is performed. Protein ionization is obligatory, but it does not lead to any changes that could enable genus identification. The next step is assessing the ionized proteins using a TOF (time of flight) analyzer, which measures the time of flight of ions that is specific for a protein with a particular molecular mass [11]. MALDI–TOF MS is fast and precise, but its sensitivity and specificity depend on the database used. Despite still-imperfect databases, the use of MALDI–TOF MS is a current standard in yeast-like fungi identification [12].

The MALDI–TOF test requires culturing. If an immediate identification is needed (e.g., due to a patient’s critical condition), it is possible to perform the test using material collected directly from the patient, but the quality of such a test is lower than when cultured fungi are used [10,13].

As far as the PCR method for fungi identification is concerned, its specificity depends on the used starters, complementary to the DNA sequences of individual fungal species. Additionally, this method allows for qualitative identification of the genetic material. RT PCR is another diagnostic tool that gives the opportunity to perform quantitative identification of the genetic material [14]. As all of the abovementioned classical methods depend on the microbiologist’s skills and experience, they seem less repeatable and objective than automated methods such as MALDI–TOF MS or PCR.

The aim of this study is to compare and verify the consistency between classical methods and MALDI–TOF MS in fungal identification.

## 2. Materials and Methods

The material was collected using a sterile transport swab set from healthy buccal mucosa from 98 adults (healthy buccal mucosa for 69 HIV individuals and healthy buccal mucosa for 29 healthy individuals). All participants signed the informed consent document, agreeing to take part in the study, and the study gained ethical approval (number: 413/2018). The study group was composed of patients of an infectious diseases clinic. Inclusion criteria to the study group were as follows: all of the participants had to be adults, HIV-infected, in antiretroviral therapy, with no visible lesions on oral cavity mucosa, and clinically stable; participants had to sign an informed consent document before the study. The control group was chosen in a way that it matched the study group in terms of sex and age; it was composed of HIV-negative individuals living in the Lower Silesia region.

The material was then cultured in a laboratory for 24 h on Sabouraud 2 with chloramphenicol (Biomerieux, France) medium. Additionally, the swab was set in a test tube with liquid Sabouraud Broth medium. The incubation was performed for 48 h at 28 °C. After this time, the colonies’ growth was assessed. The incubation was then prolonged to 7 days, and, during that time, the plates with Sabouraud medium were checked every day [7].

Next, a microscopic assessment of the cultured fungi was performed. The shape, size, color, and number of colonies were characterized. Pure single colonies were then cultured on media detecting fungal enzymatic activity—CHROMagar *Candida* (Becton Dickinson, USA)—to initially identify the species. In the case of any doubts, other methods were introduced, like rice medium Rice Agar (Biomaxima, Poland), which allows us to assess chlamydospores, pseudomycelium, and mycelium. *C. albicans* and *C. dubliniensis* produce chlamydospores, *C. glabrata* does not produce pseudomycelium, and *C. tropicalis* does not produce chlamydospores. Distinguishing *C. albicans* from *C. dubliniensis* was conducted by growing the colonies at different temperatures—37 and 42 °C*. Candida albicans* grows at 42 °C, whereas *Candida dubliniensis* grows poorly. Additionally, microscopic slides colored with lactophenol blue (Lactophenol Cotton Blue) were used to assess the micromorphology of the fungi [7,8]. All isolated strains were then analyzed by the MALDI–TOF MS method (Bruker, Poland).

## 3. Results

- In the study group (patients with HIV), positive fungal cultures were detected 66 times (95% of the sample), whereas in the control group (healthy people), only 14 times (48% of the sample).
- All 80 strains (100% of positive cultures) were tested with classical microbiological methods and with MALDI–TOF MS.
- Altogether, consistency of yeast-like fungi identification isolated from oral cavity swabs between classical microbiological methods and MALDI–TOF MS was obtained in 45 cases (~56%). The analyzed methods presented different results in 35 cases (~44%).
- Identification in the study group was compatible in half of the strains (33 strains) and incompatible in another half of the strains (33 strains) (Table 1).
- The consistency of identification between strains isolated from healthy individuals (control group) was greater. Twelve strains were identified to the same species with the classical method and MALDI–TOF, whereas only 2 strains were identified differently (Table 2).
- Ten strains from the study group were identified only to the genus level using classical methods. Six of them were identified as *C. tropicalis*, one as *C. dubliniensis*, one as *C. inconspicua*, and one as *C. parapsilosis* by MALDI–TOF MS. One strain was identified as *Pseudomonas aeruginosa* using MALDI–TOF MS.
- Among the 25 other cases, the results were different using classical methods and MALDI–TOF MS when it came to species identification. Twenty-three of them came from the study group and two from the control group. The most common difference was the identification of the strains as *C. albicans* using the classical methods and as *C. dubliniensis* using MALDI–TOF MS. This situation occurred in 20 cases: 19 from the study group and 1 from the control group. In one case, the situation was the opposite: in the control group, one strain was identified as *C. dubliniensis* by classical methods, whereas MALDI–TOF MS detected *C. albicans*.
- MALDI–TOF MS detected two strains as bacteria: the abovementioned *Pseudomonas aeruginosa* and *Rothia mucilaginosa*. In one case, MALDI–TOF MS was not able to perform the identification (Table 3).
- Colonisation by two different species of *Candida* fungi was detected in three patients using classical methods. However, during MALDI-TOF MS analysis, it turned out that the isolated fungi were of the same species, so the patients were colonized only by a single species.
- From 11 Sabouraud media plates that did not show any sign of fungal growth, three turned out to show some colony growth after multiplying the material in Sabouraud broth, a liquid medium. All of them came from the control group.

## 4. Discussion

As a relatively new diagnostic method, MALDI–TOF MS has been compared by numerous authors to standard microbiological methods [6,15,16]. It is an approved method of bacteria identification, also confirmed in other research studies [17]. Furthermore, MALDI–TOF MS has been used to identify fungi, especially yeast-like fungi. Croxatto et al. (2012) and Chao et al. (2014) have confirmed that this is a highly effective diagnostic method for this group of microorganisms [12,18]. Zhao et al. (2018), Posteraro et al. (2012), Kolecka et al. (2013), and Denis et al. (2016) have proven the quickness and precision of this method, nevertheless emphasizing the need for enlarging the databases used during the analysis, which would help prevent mistakes during the identification process [19,20,21,22].

Comparative studies on identification methods of yeast-like fungi can be found, e.g., Zhao et al. or Rath et al. However, the material used for those studies was collected from other sources, such as blood or the ear canal, potentially leading to different results caused by the characteristics of the studied microorganisms. Yeast-like fungi easily colonize the oral cavity of both HIV-negative and HIV-positive patients. Among HIV-positive patients, enlarged colonization and a higher frequency of infections can be observed. However, the strains isolated from the ear canal or blood, where they are treated as etiological factors of fungemia, can have different characteristics and phenotypic features in classical microbiological tests [19,23]. Other authors compared MALDI–TOF MS with the PCR method, which could be an effective way to verify the species analyses presented in this paper [19,24].

*C. dubliniensis* and *C. albicans* are yeast-like fungi with very similar morphological features. Therefore, the differentiation between the two using classical methods is difficult [25]. Many authors find that these species have been frequently misidentified, which can have serious clinical consequences as there are significant differences in their antibiotic susceptibility profiles [25,26]. Moreover, correct differentiation of this species is especially important for immunosuppressed patients, for example, HIV-infected patients, whose oral cavities are frequently colonized with *C. dubliniensis* [26,27]. In order to properly identify these species, it is necessary to introduce more advanced diagnostic methods that will standardize the tests using a more unified identification system, e.g., RT PCR [28].

Identification accuracy can be improved by different methods, complementing classical microbiological identification. One of them is the incubation of the fungi at different temperatures based on the fact that, unlike *C. albicans*, *C. dubliniensis* grows slowly at 42 °C and does not grow at all at 45 °C. Other authors have highlighted the improvement of identification results using the abovementioned method, but this process is not used in routine procedures, and its clinical usefulness is not known. This procedure requires further research to verify its usefulness in clinical practice because, as an inexpensive and easy to implement solution, it can largely improve the precision of classical methods [29,30,31]. Hof et al. considered the identification of fungi by MALDI–TOF MS as an effective diagnostic instrument for differentiation between *C. albicans* and *C. dubliniensis*, which was also proved in our research. In Hof’s research, incorrect identification of *C. dubliniensis* as *C. albicans* was the most frequent mistake made by classical identification methods [32].

In our paper, we have also shown a significant percentage of incorrect identifications of *C. tropicalis*, which was detected using classical methods in only one out of eight cases. In six of the cases, it was identified only to the genus. These results highlight the identification problems of this species using classical methods. Moreover, the detection of *C. tropicalis* can be important due to its different resistance profile in relation to *C. albicans*, features such as its ability to produce biofilm and its specific influence on other fungal species and their morphology and pathogenicity [33,34,35,36]. Additionally, infections caused by *C. tropicalis* have significantly higher mortality rates than those produced by other fungal species, which has also been described in the literature [37].

Most identification mistakes using classical methods occurred in the test group. Furthermore, other authors have also pointed out the uncertain identification results using classical microbiological methods in the group of immunocompromised patients [38,39]. Therefore, it is possible to draw a conclusion that fungal morphology among these patients is different and more difficult to evaluate, which worsens the results of species identification.

MALDI–TOF MS is also proposed as a quick diagnostic tool for the determination of antibiotic resistance of yeast-like fungi from the *Candida* species to, for example, fluconazole. Its application could significantly shorten the waiting time for the results compared to traditionally defined antibiotic resistance [40]. However, some authors have emphasized the difficulties of proper detection of microorganism resistance using this method, which is caused by problems in the interpretation of the obtained spectrum [41].

What is more, MALDI–TOF MS is also an efficient alternative to classical methods in terms of economic factors, as the price of one test is lower than that of classical methods. Additionally, the results are much faster, which has been emphasized by other authors. Identification of fungal species using MALDI–TOF MS takes only 10 to 15 min compared with classical methods, which can take a few days [42]. However, the equipment cost (MALDI analyzer) is very high, which explains why the method is still not commonly used. However, the advantages resulting from microorganism identification by this method are invaluable for the treatment process—faster identification and better precision of the identification method allow doctors to implement targeted therapy in a shorter period of time, possibly saving the patient.

The significant reduction in waiting time is particularly relevant for immunocompromised patients because of the risk posed by fungal infections. These infections are quite common in this population in comparison to people with healthy immune systems. Moreover, the frequent occurrence of non-*albicans* species, which are often resistant to antibiotics, is another risk factor that can lead to a serious course of infection in HIV-positive patients [43]. The MALDI–TOF MS method allows us to identify antibiotic resistance, which can further help patients with immune deficiency who are at high risk of colonization by multiresistant strains [44]. Additionally, the identification of yeast-like fungi in HIV-positive patients using traditional methods can be more difficult than among HIV-negative people, which implies a strong need to seek alternative methods of diagnosing fungal infections, such as MALDI–TOF MS, for this group of patients.

The limitations of this study were as follows: lack of CD4+ levels of the HIV-positive individuals at the time of the study; lack of quantitative assessment; no verifying methods, such as PCR; lack of some other tests, e.g., antibiotic susceptibility; finally, a limited number of participants. Each of these problems could be solved by implementing some additional procedures and should be considered by authors in their future work.

## 5. Conclusions

MALDI–TOF MS is an innovative and effective method of identification of yeast-like fungi and should be seen as an alternative to traditional microbiological methods. The crucial advantage of this method is the shortening of waiting time for the test result to approximately 10–15 min. In comparison to classical methods (in which results are available after min. 24–48 h), it allows doctors to make a quick decision on which antifungal treatment to use. However, this method is not free from drawbacks. Thus, a constant extension of the databases used to identify microorganisms is required.

## Figures and Tables

**Figure 1:**
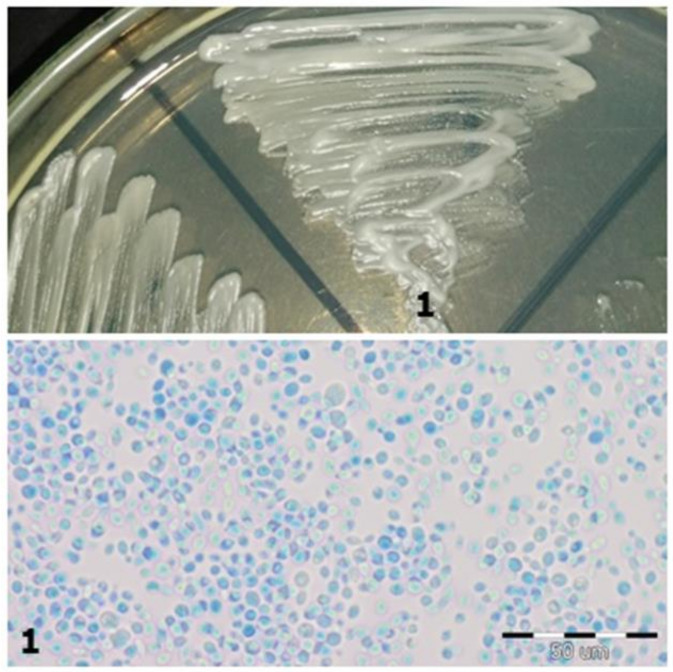
Strain 1-morphology on Sabouraud 2 with chloramphenicol medium and 40× zoom (Lactophenol Cotton Blue; private collection).

**Figure 2:**
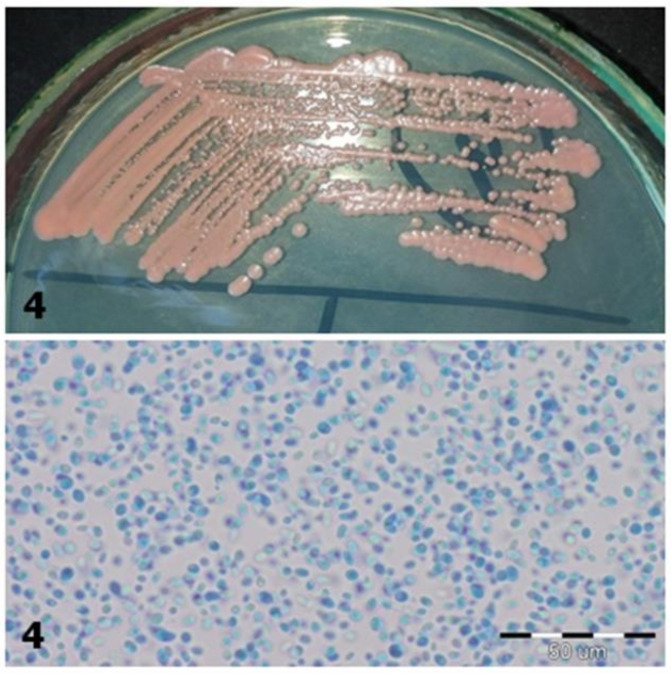
Strain 4-morphology on Sabouraud 2 with chloramphenicol medium and 40× zoom (Lactophenol Cotton Blue; private collection).

**Figure 3:**
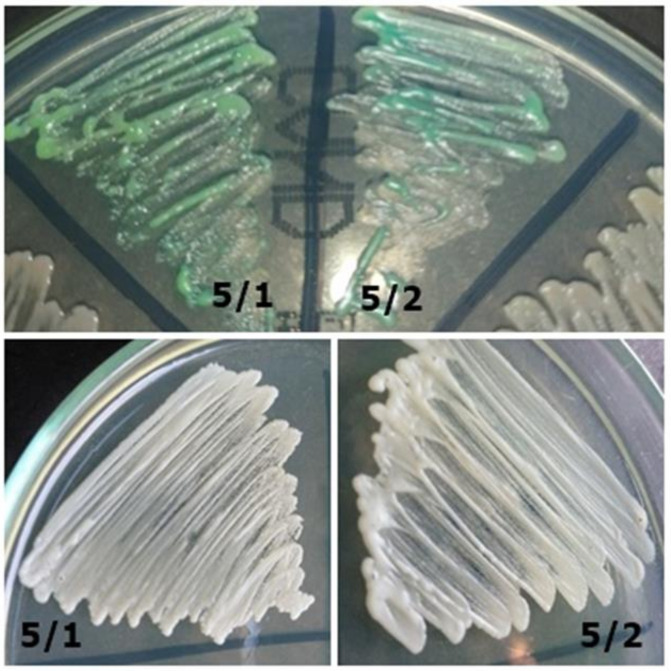
Strains 5/1 and 5/2-morphology on CHROMagar *Candida* and Sabouraud 2 with chloramphenicol medium (private collection).

**Figure 4:**
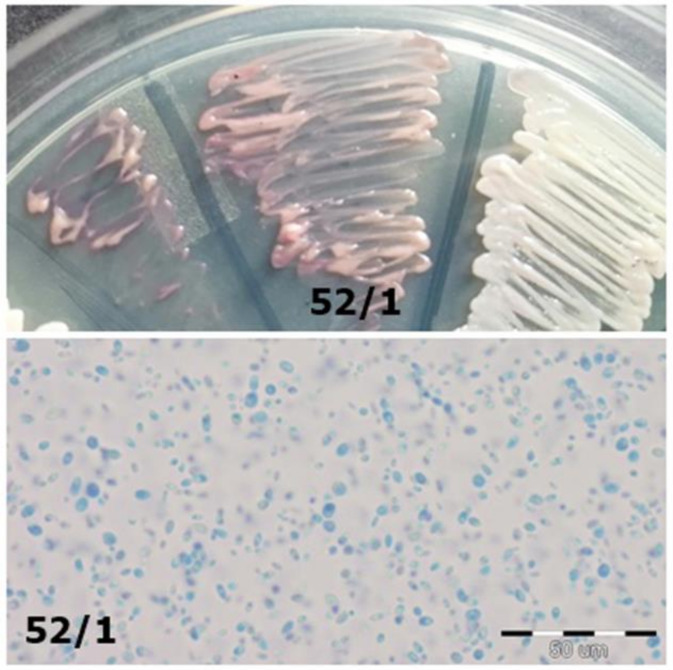
Strain 52/1-morphology on CHROMagar *Candida* and 40× zoom (Lactophenol Cotton Blue; private collection).

**Figure 5:**
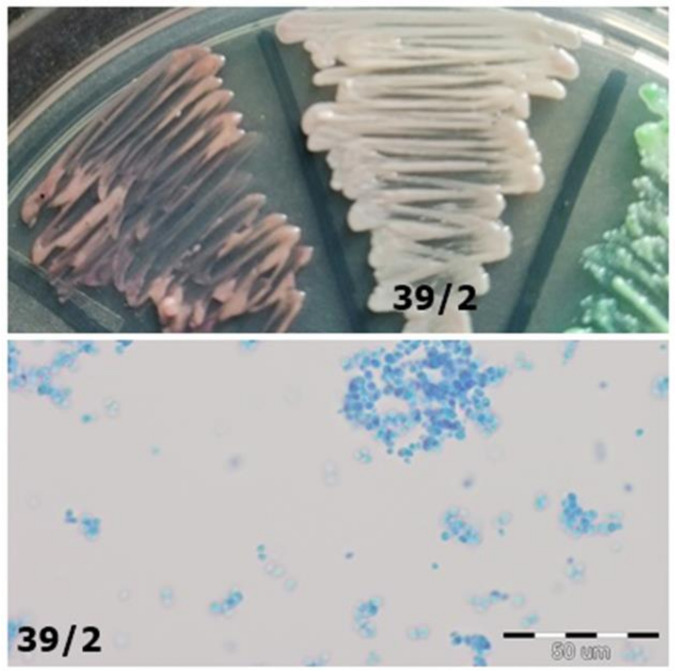
Strain 39/2-morphology on CHROMagar *Candida* and 40× zoom (Lactophenol Cotton Blue; private collection).

**Figure 6:**
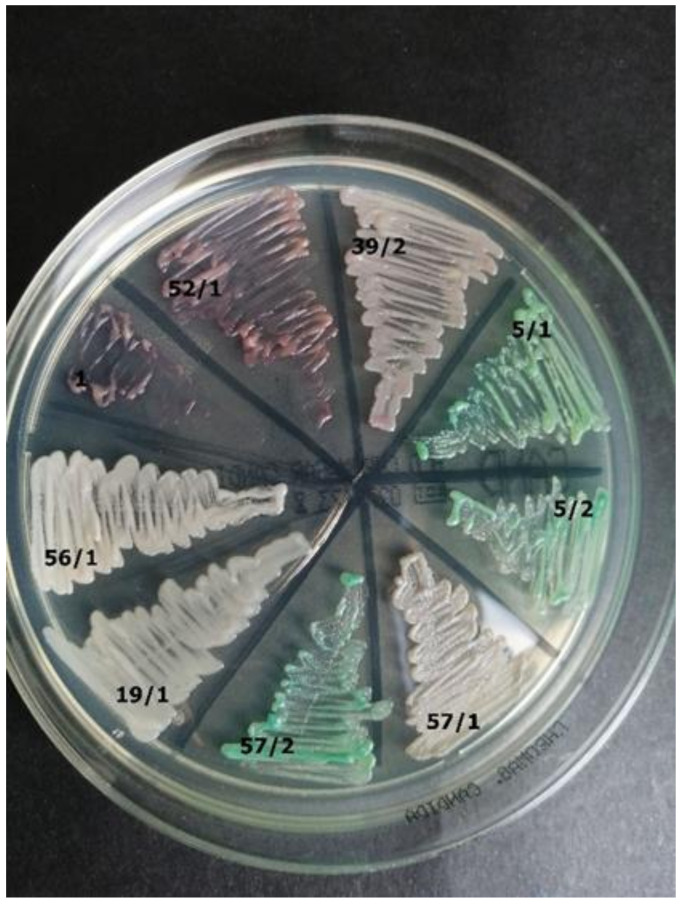
Identification of yeast-like fungi (Table 3) on CHROMagar *Candida* (private collection).

**Figure 7:**
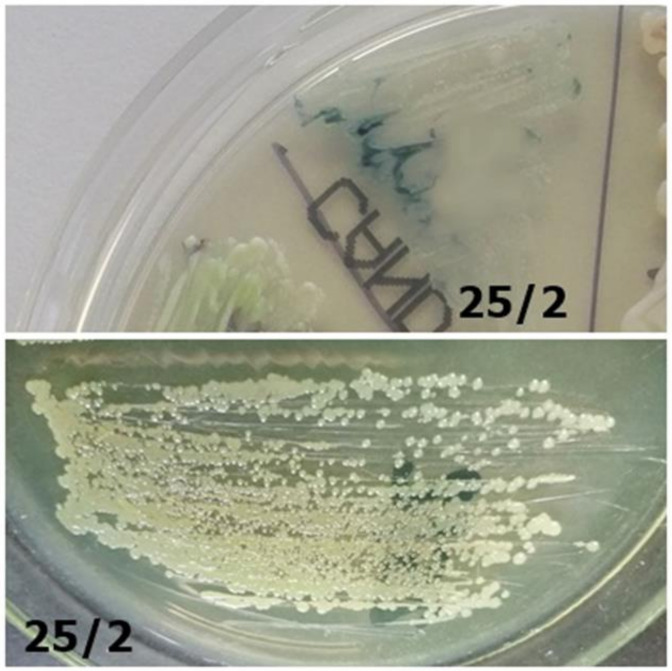
Strain 25/2-morphology on CHROMagar *Candida* and Sabouraud 2 with chloramphenicol medium (private collection).

**Figure 8:**
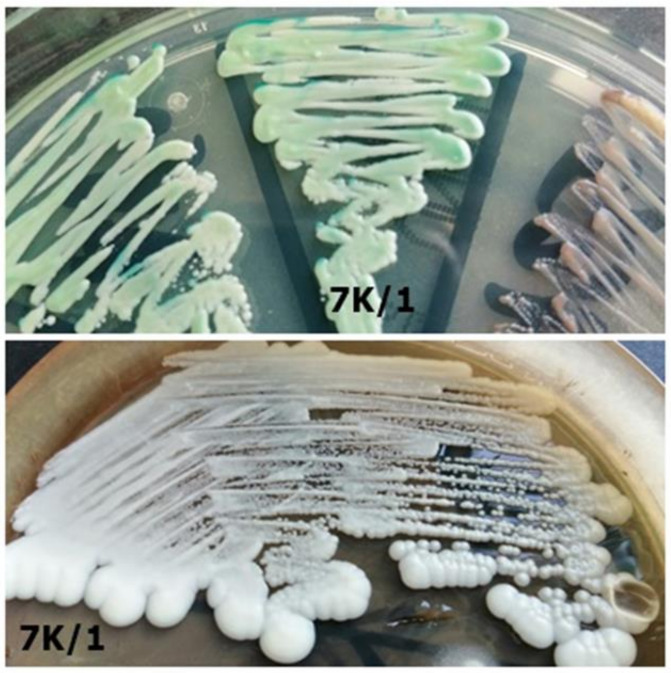
Strain 7K/1-morphology on CHROMagar *Candida* and Sabouraud 2 with chloramphenicol medium (private collection).

**Table 1:** Comparison of results of fungi identification in the study group (people infected with HIV).

Study Group/Patients with HIV	Number of Strains		Classical Methods	MALDI–TOF MS
	Total	Single		
Consistent results	33	20	*C. albicans*	*C. albicans*
		5	*C. dubliniensis*	*C. dubliniensis*
		6	*C. glabrata*	*C. glabrata*
		1	*C. krusei*	*C. krusei*
		1	*C. tropicalis*	*C. tropicalis*
Inconsistent results	33	19	*C. albicans*	*C. dubliniensis*
		1	*C. albicans*	*C. tropicalis*
		1	*C. glabrata*	*Cryptococcus laurenti*
		1	*C. glabrata*	*C. albicans*
		6	*Candida spp.*	*C. tropicalis*
		1	*Candida spp.*	*C. dubliniensis*
		1	*Candida spp.*	*C. inconspicua*
		1	*Candida spp.*	*C. parapsilosis*
		1	*Candida spp.*	*Pseudomonas fluorescens*
		1	*Rhodothorula*	*Rothia mucilaginosa*

**Table 2:** Comparison of results of fungi identification in the control group (healthy people, not infected with HIV).

Control Group/Healthy Individuals	Number of Strains		Classical Methods	MALDI–TOF MS
	Total	Single		
Consistent results	12	5	*C. albicans*	*C. albicans*
		6	*C. dubliniensis*	*C. dubliniensis*
		1	*C. glabrata*	*C. glabrata*
Inconsistent results	2	1	*C. albicans*	*C. dubliniensis*
		1	*C. dubliniensis*	*C. albicans*

**Table 3:** Comparison of identification results between selected strains from study and control group—differences in strain identification by the two methods.

Strain No.	Classical Method—Plate Method CHromAgar/Sabouraud Agar	MALDI–TOF MS	Comments
HIV-positive patients			
1	*C. glabrata*	*Cryptococcus laurentii*	Violet on the CHROMagar substrate/plate In the preparation with Lactophenol, the cells are round, ovate (Figure 1)
4	*Rhodotorula spp.*	*Rothia mucilaginosa*	Coral color on Sabouraud Agar; In the preparation with Lactophenol, round, ellipsoidal cells with a size of 3.5–6.5 µm, proving that they are fungal cells (Figure 2)
5/1	*Candida sp.*	*Candida dubliniensis*	Creamy matte on a Sabouraud Agar; on ChROMagar, green (lighter than in 5/2 strain) (Figure 3)
5/2	*C. albicans*	*Candida dubliniensis*	Shiny white on Sabouraud Agar; on CHROMagar, green medium (darker than in the 5/1 strain) (Figure 3)
52/1	*C. glabrata*	Problems with identification	Violet on ChromAgar In the preparation with Lactophenol, cells similar to *Candida glabrata* (Figure 4)
39/2	*C. glabrata*	*C. albicans*	Iridescent with purple edges, like *C.* glabrata; it is not green, as is the case with *C. albicans* (Figure 5)
57/1	*Candida spp.*	*C. tropicalis*	No clear color on the CHROMagar; there is also no sea color, dark blue, or gray-blue to indicate the presence of *Candida tropicalis* on CHROMagar (Figure 6)
57/2	*C. albicans*	*C. dubliniensis*	Green on CHROMagar (Figure 6)
56/1	*Candida spp.*	*C. incospicua*	Shiny white on CHROMagar (Figure 6)
19/1	*Candida spp.*	*C. parapsilosis*	White on CHROMagar (Figure 6)
25/2	Problems with identification	*Pseudomonas fluorescens*	Poor/inhibition growth on CHROMagar; opalescent on Sabouraud agar (Figure 7)
Healthy patients			
7K/1	*Candida albicans*	*Candida albicans*	Light green on CHROMagar; Shiny white on Sabouraud agar (Figure 8)

## Data Availability

All data generated or analyzed during this study are included in this published article.

## References

[1] Neppelenbroek K.H., Seó R.S., Urban V.M., Silva S., Dovigo L.N., Jorge J.H., Campanha N.H. (2014). Identification of Candida species in the clinical laboratory: A review of conventional, commercial, and molecular techniques. Oral Dis..

[2] Maldonado I., Cataldi S., Garbasz C., Relloso S., Striebeck P., Guelfand L., López Moral L. (2018). Identification of Candida yeasts: Conventional methods and MALDI-TOF MS. Rev. Iberoam. Micol..

[3] Almeida-Silva F., Damasceno L.S., Serna M.J., Valero C., Quintella L.P., Almeida-Paes R., Muniz Mde M., Zancope-Oliveira R.M. (2016). Multiple opportunistic fungal infections in an individual with severe HIV disease: A case report. Rev. Iberoam. Micol..

[4] Vargas K., Messer S.A., Pfaller M., Lockhart S.R., Stapleton J.T., Hellstein J., Soll D.R. (2000). Elevated Phenotypic Switching and Drug Resistance of *Candida albicans* from Human Immunodeficiency Virus-Positive Individuals prior to First Thrush Episode. J. Clin. Microbiol..

[5] Dingle T.C., Butler-Wu S.M. (2013). Maldi-tof mass spectrometry for microorganism identification. Clin. Lab. Med..

[6] Ibáñez-Martínez E., Ruiz-Gaitán A., Pemán-García J. (2017). Update on the diagnosis of invasive fungal infection. Rev. Esp. Quimioter..

[7] Krzyściak P., Skóra M., Macura A.B. (2010). Atlas Grzybów Chorobotwórczych Człowieka.

[8] Nawrot U., Włodarczyk K., Skała J., Przondo-Mordarska A., Nolard N. (2005). Ocena przydatności chromogennego podłoża CandiSelect 4(BioRad) do izolacji i wstępnej identyfikacji grzybów z rodzaju Candida. Mikol. Lek..

[9] Kawalec A., Białecka A., Kasprowicz A., Barabasz W. (2016). Identyfikacja grzybów drożdżopodobnych izolowanych z dróg rodnych kobiet z wykorzystaniem spektrometrii mas typu MALDI-TOF. Med. Dośw. Mikrobiol..

[10] Hou T.Y., Chiang-Ni C., Teng S.H. (2019). Current status of MALDI-TOF mass spectrometry in clinical microbiology. J. Food Drug Anal..

[11] Singhal N., Kumar M., Kanaujia P.K., Virdi J.S. (2015). MALDI-TOF mass spectrometry: An emerging technology for microbial identification and diagnosis. Front. Microbiol..

[12] Croxatto A., Prod’hom G., Greub G. (2012). Applications of MALDI-TOF mass spectrometry in clinical diagnostic microbiology. FEMS Microbiol. Rev..

[13] Rodríguez-Sánchez B., Cercenado E., Coste A.T., Greub G. (2019). Review of the impact of MALDI-TOF MS in public health and hospital hygiene, 2018. Euro Surveill..

[14] Deepak S., Kottapalli K., Rakwal R., Oros G., Rangappa K., Iwahashi H., Masuo Y., Agrawal G. (2007). Real-Time PCR: Revolutionizing Detection and Expression Analysis of Genes. Curr. Genomics.

[15] Oviaño M., Rodríguez-Sánchez B. (2020). MALDI-TOF mass spectrometry in the 21st century clinical microbiology laboratory. Enferm. Infecc. Microbiol. Clin..

[16] Sow D., Fall B., Ndiaye M., Ba B.S., Sylla K., Tine R., Lô A.C., Abiola A., Wade B., Dieng T. (2015). Usefulness of MALDI-TOF Mass Spectrometry for Routine Identification of Candida Species in a Resource-Poor Setting. Mycopathologia.

[17] Carbonelle E., Mesquita C., Bille E., Day N., Dauphin B., Beretti J.L., Ferroni A., Gutmann L., Nassif X. (2011). MALDI-TOF mass spectrometry tools for bacterial identification in clinical microbiology laboratory. Clin. Biochem..

[18] Chao Q.T., Lee T.F., Teng S.H., Peng L.Y., Chen P.H., Teng L.J., Hsueh P.R. (2014). Comparison of the accuracy of two conventional phenotypic methods and two MALDI-TOF MS systems with that of DNA sequencing analysis for correctly identifying clinically encountered yeasts. PLoS ONE.

[19] Zhao Y., Tsang C.C., Xiao M., Chan J.F.W., Lau S.K.P., Kong F., Xu Y., Woo P.C.Y. (2018). Yeast identification by sequencing, biochemical kits, MALDI-TOF MS and rep-PCR DNA fingerprinting. Med. Mycol..

[20] Posteraro B., Vella A., Cogliati M., De Carolis E., Florio A.R., Posteraro P., Sanguinetti M., Tortorano A.M. (2012). Matrix-assisted laser desorption ionization-time of flight mass spectrometry-based method for discrimination between molecular types of *Cryptococcus neoformans* and *Cryptococcus gattii*. J. Clin. Microbiol..

[21] Kolecka A., Khayhan K., Groenewald M., Theelen B., Arabatzis M., Velegraki A., Kostrzewa M., Mares M., Taj-Aldeen S.J., Boekhout T. (2013). Identification of medically relevant species of arthroconidial yeasts by use of matrix-assisted laser desorption ionization-time of flight mass spectrometry. J. Clin. Microbiol..

[22] Denis J., Machouart M., Morio F., Sabou M., Kauffmann-LaCroix C., Contet-Audonneau N., Candolfi E., Letscher-Bru V. (2016). Performance of Matrix-Assisted Laser Desorption Ionization-Time of Flight Mass Spectrometry for Identifying Clinical Malassezia Isolates. J. Clin. Microbiol..

[23] Rath S., Das S.R., Padhy R.N. (2019). Bayesian analysis of two methods MALDI-TOF-MS system and culture test in otomycosis infection. World J. Otorhinolaryngol. Head Neck Surg..

[24] Pulcrano G., Iula D.V., Vollaro A., Tucci A., Cerullo M., Esposito M., Rossano F., Catania M.R. (2013). Rapid and reliable MALDI-TOF mass spectrometry identification of Candida non-albicans isolates from bloodstream infections. J. Microbiol. Methods.

[25] Gutiérrez J., Morales P., González M.A., Quindós G. (2002). *Candida dubliniensis*, a new fungal pathogen. J. Basic Microbiol..

[26] Momani O.M., Qaddoomi A. (2005). Identification of *Candida dubliniensis* in a diagnostic microbiology laboratory. East Mediterr. Health J..

[27] Paugam A., Baixench M.T., Viguié C. (2008). Actualités sur *Candida dubliniensis* [An update on *Candida dubliniensis*]. Med. Mal. Infect..

[28] Asadzadeh M., Ahmad S., Al-Sweih N., Khan Z. (2018). Rapid and Accurate Identification of *Candida albicans* and *Candida dubliniensis* by Real-Time PCR and Melting Curve Analysis. Med. Princ. Pract..

[29] Tintelnot K., Haase G., Seibold M., Bergmann F., Staemmler M., Franz T., Naumann D. (2000). Evaluation of phenotypic markers for selection and identification of *Candida dubliniensis*. J. Clin. Microbiol..

[30] Mesa L.M., Arcaya N., Cañas O., Machado Y., Calvo B. (2004). Evaluación de los caracteres fenotípicos para diferenciar Candida Albicans de *Candida dubliniensis* [Phenotypic evaluation to differentiate *Candida albicans* from *Candida dubliniensis*]. Rev. Iberoam. Micol..

[31] Odds F.C., Davidson A. (2000). “Room temperature” use of CHROMagar Candida. Diagn. Microbiol. Infect. Dis..

[32] Hof H., Eigner U., Maier T., Staib P. (2012). Differentiation of *Candida dubliniensis* from *Candida albicans* by means of MALDI-TOF mass spectrometry. Clin. Lab..

[33] de Barros P.P., Rossoni R.D., Freire F., Ribeiro F.C., Lopes L.A.D.C., Junqueira J.C., Jorge A.O.C. (2018). Candida tropicalis affects the virulence profile of Candida albicans: An in vitro and in vivo study. Pathog. Dis..

[34] Negri M., Silva S., Breda D., Henriques M., Azeredo J., Oliveira R. (2012). *Candida tropicalis* biofilms: Effect on urinary epithelial cells. Microb. Pathog..

[35] Silva S., Negri M., Henriques M., Oliveira R., Williams D.W., Azeredo J. (2012). Candida glabrata, Candida parapsilosis and Candida tropicalis: Biology, epidemiology, pathogenicity and antifungal resistance. FEMS Microbiol. Rev..

[36] Xisto M.I., Caramalho R.D., Rocha D.A., Ferreira-Pereira A., Sartori B., Barreto-Bergter E., Junqueira M.L., Lass-Flörl C., Lackner M. (2017). Pan-azole-resistant Candida tropicalis carrying homozygous erg11 mutations at position K143R: A new emerging superbug?. J. Antimicrob. Chemother..

[37] Arastehfar A., Daneshnia F., Hafez A., Khodavaisy S., Najafzadeh M.J., Charsizadeh A., Zarrinfar H., Salehi M., Shahrabadi Z.Z., Sasani E. (2020). Antifungal susceptibility, genotyping, resistance mechanism, and clinical profile of *Candida tropicalis* blood isolates. Med. Mycol..

[38] Lomeli-Martinez S.M., Valentin-Goméz E., Varela-Hernández J.J., Alvarez-Zavala M., Sanchez-Reyes K., Ramos-Solano M., Cabrera-Silva R.I., Ramirez-Anguiano V.M., Lomeli-Martinez M.A., Martinez-Salazar S.Y. (2019). *Candida* spp. Determination and Th1/Th2 Mixed Cytokine Profile in Oral Samples From HIV+ Patients With Chronic Periodontitis. Front. Immunol..

[39] Paul S., Kannan I. (2019). Molecular identification and antifungal susceptibility pattern of *Candida* species isolated from HIV infected Patients with candisiasis. Curr. Med. Mycol..

[40] Delavy M., Cerutti L., Croxatto A., Prod’hom G., Sanglard D., Greub G., Coste A.T. (2020). Machine Learning Approach for *Candida albicans*Fluconazole Resistance Detection Using Matrix-Assisted Laser Desorption/Ionization Time-of-Flight Mass Spectrometry. Front. Microbiol..

[41] Brackmann M., Leib S.L., Tonolla M., Schürch N., Wittwer M. (2020). Antimicrobial resistance classification using MALDI-TOF-MS is not that easy: Lessons from vancomycin-resistant *Enterococcus faecium*. Clin. Microbiol. Infect..

[42] UNC Finds Cost Of MALDI-TOF System Is Offset In 3 Years. https://www.rapidmicrobiology.com/news/maldi-tof-identification-system-cost-offset-3-years.

[43] Maheshwari M., Kaur R., Chadha S. (2016). Candida Species Prevalence Profile in HIV Seropositive Patients from a Major Tertiary Care Hospital in New Delhi, India. J. Pathog..

[44] Paul S., Singh P., Rudramurthy S.M., Chakrabarti A., Ghosh A.K. (2018). Rapid detection of fluconazole resistance in *Candida tropicalis* by MALDI-TOF MS. Med. Mycol..

